# The FGF23–Klotho axis and cardiac tissue Doppler imaging in pediatric chronic kidney disease—a prospective cohort study

**DOI:** 10.1007/s00467-017-3766-5

**Published:** 2017-08-09

**Authors:** Ylva Tranæus Lindblad, Hannes Olauson, Georgios Vavilis, Ulf Hammar, Maria Herthelius, Jonas Axelsson, Peter Bárány

**Affiliations:** 1 Division of Pediatrics, Department of Clinical Sciences, Intervention and Technology (CLINTEC), Karolinska Institutet, Stockholm, Sweden; 2 Astrid Lindgren Children’s Hospital, Huddinge BUMM, Paradistorget 4, 5tr, S-141 47 Huddinge, Sweden; 3 Department of Pediatrics, Karolinska University Hospital, Stockholm, Sweden; 4 Division of Renal Medicine, CLINTEC, Karolinska Institutet, Stockholm, Sweden; 5 Division of Emergency Medicine, Karolinska University Hospital, Stockholm, Sweden; 6 Institute of Environmental Medicine and Unit of Biostatistics, Karolinska Institutet, Stockholm, Sweden; 7 Department of Immunology, Karolinska University Hospital, Stockholm, Sweden; 8 Department of Medical Biochemistry and Biophysics, Karolinska Institutet, Stockholm, Sweden; 9 Department of Renal Medicine, Karolinska University Hospital, Stockholm, Sweden

**Keywords:** Chronic kidney disease, Kidney transplantation, Pediatrics, Fibroblast growth factor 23, Klotho, Cardiovascular diseases

## Abstract

**Background:**

Chronic kidney disease-associated mineral bone disorder (CKD-MBD) is common in pediatric kidney disease patients and a risk factor for future cardiovascular disease (CVD). Fibroblast growth factor-23 (FGF23) and Klotho are novel key players in CKD-MBD, and has been suggested to be involved in the development of CVD.

**Methods:**

This prospective cohort study included 74 pediatric patients; 31 with CKD (age range 0.8–18.8 years, glomerular filtration rate (GFR) range 9–68 mL/min/1.73 m^2^) and 43 transplanted patients (CKD-T; age range 3.3–17.7 years, GFR range 10–99 mL/min/1.73 m^2^) examined annually for 3 years. We assessed longitudinal patterns and predictors of FGF23 and soluble Klotho, as well as associations to cardiac remodeling and function using echocardiographic pulse wave Doppler (PWD) and color-coded tissue Doppler imaging (cc-TDI).

**Results:**

The prevalence of high FGF23 levels (≥95th percentile) was 60% in CKD and 42% in CKD-T patients, despite a low prevalence of hyperphosphatemia and normal Klotho levels. Low GFR at baseline was a predictor for high mean log FGF23 during follow-up in CKD and CKD-T patients (β = −0.2, *p* < 0.001). A high log FGF23* z*-score longitudinally was borderline significantly associated with elevated left ventricular mass index (LVMI) in CKD patients (β = 1.8, *p* = 0.06). In addition, high log FGF23 (β = −0.43, *p* = 0.01) and low log Klotho (β = 0.44, *p* = 0.006) over time were associated with a worse left ventricular diastolic function (cc-TDI e′/a′) in CKD-T patients.

**Conclusions:**

In pediatric CKD and CKD-T patients, the FGF23 level increase and Klotho level decrease with progressing renal failure, despite well-controlled phosphate levels. Following adjustments, both high FGF23 and low Klotho levels were strongly associated with a worse left ventricular diastolic function longitudinally. The potential role of FGF23 and Klotho in cardiac morbidity in pediatric CKD requires further investigation.

**Electronic supplementary material:**

The online version of this article (doi:10.1007/s00467-017-3766-5) contains supplementary material, which is available to authorized users.

## Introduction

Cardiovascular disease (CVD) represents one of the most important causes of death in pediatric chronic kidney disease (CKD) patients [1]. CKD-associated mineral bone disorder (CKD-MBD) contributes substantially to the increased cardiovascular risk in this patient group [2]. The bone-derived fibroblast growth factor-23 (FGF23) is a novel marker of CKD-MBD, that increase progressively with declining renal function [3–10] and markedly increase in end-stage renal disease (ESRD) [6, 10, 11]. While FGF23 levels decrease following renal transplantation, they are still elevated compared with healthy individuals [6, 10, 12, 13].

FGF23 regulates phosphate and vitamin D balance by inducing urinary phosphate excretion in the proximal tubule, and it also inhibits the conversion of 25-hydroxy vitamin D to its active form. The actions of FGF23 are mediated through FGF-receptors (FGFRs) with the aid of its co-receptor α-Klotho (Klotho) [14]. Klotho is a membrane-bound protein predominantly expressed in the kidney, parathyroid gland and choroid plexus. It is also shed from the cell surface by α-secretases, acting in its soluble form independent of FGF23.

In recent years, a growing body of evidence points to FGF23 as a novel predictor of mortality in adult CKD [15–19]. The association between FGF23 and mortality is mainly attributed to the increased risk of cardiovascular events, but also due to progression of CKD in itself. Specifically, FGF23 is associated with left ventricular hypertrophy (LVH) [19–21], impaired left ventricular function [19], endothelial dysfunction [22, 23], heart failure [24] and progression of renal failure [15, 19] in adult CKD. Recent experimental data suggest that FGF23 can induce LVH directly by activating FGFR4 in cardiomyocytes [25]. Further, soluble Klotho has been shown to have protective effects on the cardiovascular system by preventing endothelial dysfunction, vascular calcifications [26], cardiac fibrosis and cardiac hypertrophy [27].

Increased FGF23 levels are reported in the majority of pediatric patients with CKD, but most studies performed to date are cross-sectional, making it difficult to draw conclusions on predictors and consequences. Indeed, very few studies have analyzed longitudinal patterns of FGF23 and soluble Klotho in pediatric CKD patients or renal transplant recipients [6, 13, 28]. Also, studies on associations between the FGF23–Klotho axis and CVD in pediatric CKD are scarce [29] [8].

The objectives of this study were to: (1) analyze longitudinal patterns of FGF23 and soluble Klotho, (2) examine predictors of FGF23 and soluble Klotho levels and (3) investigate possible associations between FGF23 and soluble Klotho to surrogate measures of CVD, in a cohort of pediatric non-dialysis CKD patients and renal transplant recipients followed prospectively for 3 years.

## Subjects and methods

### Study population and design

**Fig. 1:**
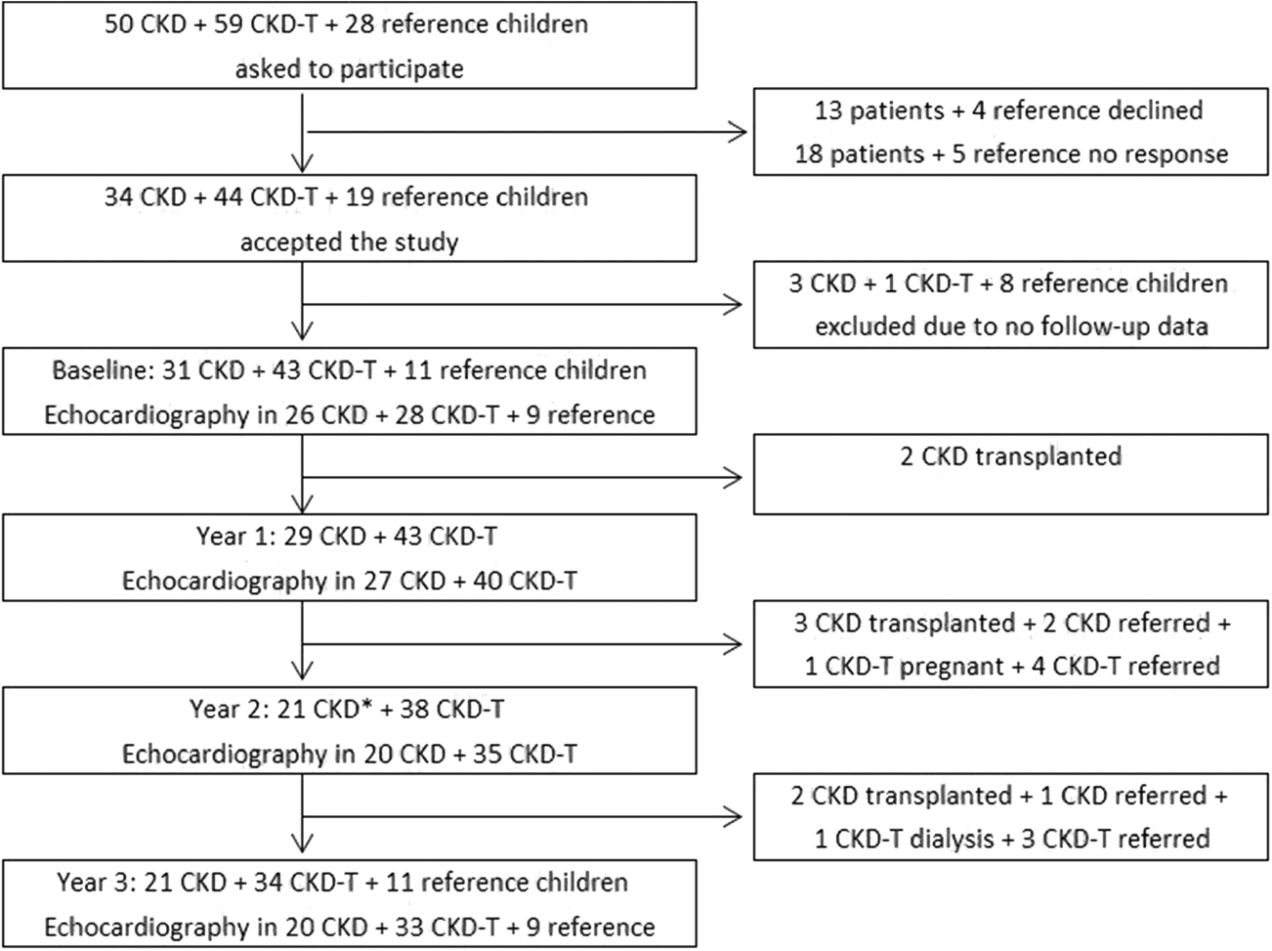
Flow chart of the study population and reasons for drop-out. The study cohort comprised children with chronic kidney disease (CKD), i.e. those with non-dialysis CKD stage 2–5 (*CKD*) and renal transplant recipients (*CKD-T*), and reference children. *Asterisk* In year 2 there were 3 patients with CKD for whom follow-up data were missing.

The study design and results from a cross-sectional analysis of the cohort at the year of inclusion have previously been published [30]. The study was designed as an observational prospective cohort study of children with CKD, either non-dialysis CKD stage 2–5 patients (CKD) or renal transplant recipients (CKD-T). All patients were treated at the outpatient Pediatric Nephrology Clinic at Astrid Lindgren Children’s Hospital, Karolinska University Hospital Huddinge in Sweden, with recruitment taking place between 2007 and 2008. The final study population consisted of 31 CKD and 43 CKD-T patients and 11 reference children. The patients were seen annually for 3 years (4 occasions), with incomplete follow-up in three CKD patients and nine CKD-T patients for whom the reasons are listed in Fig. 1. Further, seven CKD patients were transplanted during the follow-up period. This adds up to a total of 19 drop-outs.

### Biochemical characterization

Klotho, FGF23, and calcium, phosphate, intact parathyroid hormone (i-PTH), hemoglobin, high-sensitivity C-reactive protein, lipid profile (triglyceride, cholesterol, low-density lipoprotein, high-density lipoprotein), insulin and glucose (all important CKD and CVD biomarkers) were analyzed in all study participants. Blood was drawn in a standardized manner during a clinical visit in the morning and following an overnight fast. Hypercalcemia was defined as an albumin-adjusted calcium level of >2.6 mmol/L in children aged 1–17 years and >2.5 mmol/L when 18 years or older. Hyperphosphatemia was defined as a phosphate level of >1.8 mmol/L at ages 1–3 years, >2.0 mmol/L at ages 4–10 years and >1.6 mmol/L at ages 11–17 years; for patients aged 18 years and older, the cutoff was >1.5 mmol/L for females and >1.6 mmol/L for males. Secondary hyperparathyroidism was defined as an i-PTH level of >65 ng/L.

FGF23, Klotho and insulin were analyzed after serum and plasma had been stored at −80 °C. C-terminal FGF23 (RU/mL) was analyzed in EDTA plasma with a second generation human sandwich enzyme-linked immunosorbent assay (ELISA) (Immutopics, Inc., San Clemente, CA), and age adjusted* z*-scores for FGF23 were assessed [31]. Human soluble α-Klotho (pg/ml) was analyzed in serum with a solid-phase sandwich ELISA (Immuno-Biological Laboratories, Fujioka-Shi, Japan). Insulin (μIU/ml) was analyzed using the Immulite 1000 Immunoassay System (Siemens Healthcare Diagnostics AB, Upplands Väsby, Sweden). Insulin resistance was defined using the homeostasis model assessment index [32]. Records of albuminuria assessed in early morning spot urine were also collected, and significant albuminuria was defined as an urinary albumin level of ≥20 mg/L. The glomerular filtration rate (GFR) was assessed by using iohexol- or inulin-clearances in the majority of the assessments (86%), and estimated from cystatin C or creatinine levels [33] in the remaining 9 and 5% of patients, respectively (Table 1).

### Echocardiographic examination

Echocardiographic data were available for 26 CKD and 28 CKD-T patients (73%) at baseline, 27 CKD and 40 CKD-T patients (95.8%) at year 1, 20 CKD and 35 CKD-T patients (93.2%) at year 2 and 20 CKD and 33 CKD-T patients (96.4%) at year 3 (Fig. 1). The echocardiographic examinations were carried out using a standard system (Vivid 7, GE VingMed Ultrasound, Horten, Norway). A two-dimensional guided M-mode measurement, conventional pulse wave Doppler (PWD) and color-coded tissue Doppler imaging (cc-TDI) were performed according to the American Society of Echocardiography guidelines [34, 35]. Left ventricular mass index (LVMI) was assessed (left ventricular mass/height^2.7^) [34] [36]. Left ventricular diastolic function was evaluated with cc-TDI analyzing the peak myocardial velocities (cm/s) during early (e′) and late (a′) diastole. The mean velocities of the septal and lateral margins of the mitral annulus were assessed as well as the e′/a′ ratio, according to recommendations [35]. The diastolic function was also assessed by PWD measuring mitral inflow velocity in early (E) diastole [37] and the PWD E/TDI e′ ratio was calculated [38]. Details on the methods used, longitudinal changes and intra-observer variability of these echocardiographic analyses have been published recently [39].

### Statistical analysis

Statistical analyses were performed using Stata version 12.0 (StataCorp LP, College Station, TX). Results are expressed as the mean ± standard deviation and the median with the range. Univariate analyses were performed using analysis of variance (ANOVA) or Kruskal–Wallis test for comparisons between the three groups. All log-transformations were made using base “e”.

To account for the exponential association between log FGF23 and GFR, we used linear regression using restricted cubic splines with three knots, with different knots for CKD and CKD-T patients. For univariate and multivariate longitudinal analyses, we used linear mixed models, including a random subject effect, which takes into account that a subject is measured several times.

We used both raw data and calculated* z*-scores for FGF23 analyses with cutoffs for high levels set at >101 RU/mL [6] or ≥95th percentile [31]. As* z*-score reference data are not available for Klotho, we used FGF23 raw data when comparing or combining analyses for Klotho and FGF23.

The primary outcomes in the linear mixed model were FGF23 (log RU/ml) and Klotho (log pg/ml). Model 1 included both baseline and follow-up measurements for the independent variables (assessing associations), while model 2 included only baseline measurements (analyzing prediction).

The secondary outcomes were markers of cardiovascular morbidity, i.e. LVMI, cc-TDI e/a′ and the PWD E/TDI e′ ratio. Variables with *p* values of <0.10 in the univariate models were incorporated into the multivariable models. Due to potential confounding, age at baseline (years), body mass index (BMI) and systolic blood pressure (SBP)* z*-scores as well as GFR (ml/min/1.73 m^2^) were forced into the model. A *p* value of <0.05 was considered to be statistically significant.

Subjects initiating renal replacement therapy (dialysis or kidney transplant) during the study were considered to be dropouts, resulting in potential bias due to nonrandom missing values, as subjects with the most advanced disease were lost to follow-up. To estimate the immediate effect of transplantation, CKD patients referred for renal transplantation during the study were analyzed separately using a paired Wilcoxon test.

## Results

### Demographics and clinical characteristics at baseline

The baseline clinical characteristics of 31 CKD and 43 CKD-T patients as well as 11 reference children are summarized in Table 1. Figure 1 shows patient flows throughout the study. As expected, the distributions across CKD stages varied between CKD and CKD-T patients, with no CKD patients but 4 (﻿9%) CKD-T patients at stage 1; 5 (16%) CKD patients and 15 (﻿35%) CKD-T patients at stage 2; 11 (﻿35%) CKD patients and 23 (﻿53%) CKD-T patients at stage 3; 11 (﻿35%) CKD patients and no CKD-T patient at stage 4; 4 (13%) CKD patients and one (2%) CKD-T patient at stage 5. There was no significant difference in mean time of follow-up in CKD and CKD-T patients as well as in the reference children (*p* = 0.08). Overall, 10 (32.3%) CKD and 9 (20.9%) CKD-T patients dropped out during follow-up, with the reasons listed in Fig. 1. All 11 reference children completed the first and final follow-up.

**Table 1:** Baseline characteristics of the study population

Variables	Study cohort (*n* = 85 children)^a^			*p* value
	Reference group (*n* = 11	CKD group (*n* = 31)	CKD-T group (*n* = 43)	
Age (year)	10.2 [4.4–17.7]	9.8 [0.80–18.8]	13.6 [3.3–17.7]	0.06
Male	6 (54.5%)	20 (64.5%)	23 (53.5%)	0.62
Duration of CKD (in years)		4.6 [0.80–14.5]	10.9 [1.8–17.4]	<0.001*
Time after transplantation (in years)			5.0 [0.92–16.3]	
Duration of follow-up (in years)	3.3 ± 0.59	3.1 ± 0.71	2.8 ± 0.78	0.08
BMI (*z*-score)	0.21 ± 1.0	0.15 ± 1.2	0.76 ± 1.0	0.04*^d^
Overweight	2 (18.2%)	3 (9.7%)	9 (20.9%)	
Obese	0	1 (3.2%)	3 (7.0%)	
GFR (ml/min/1.73 m^2^)	107 [97–133]	30 [8.8–68]	55 [10–99]	<0.001*^e^
Office blood pressure (*z*-score)				
Systolic blood pressure	0.62 ± 0.62	0.66 ± 1.2	0.70 ± 0.97	0.90
Diastolic blood pressure	0.17 ± 0.53	0.59 ± 0.90	0.43 ± 0.82	0.39
Office hypertension	0	7 (22.6%)	9 (20.9%)	
Albuminuria^b^	0	19 (61.3%)	15 (34.9%)	
Medications				
ACEs and/or ARBs	0	15 (48.4%)	18 (41.9%)	
Antihypertensives	0	16 (51.6%)	24 (54.5%)	
Corticosteroids	0	2 (6.5%)	43 (100%)	
Immunosuppressives^c^	0	2 (6.5%)	43 (100%)	
Calcitriol or alfacalcidol	0	13 (41.9%)	5 (11.6%)	
Calcium carbonate	0	13 (41.9%)	5 (11.6%)	
Sevelamer	0	4 (12.9%)	0	
Growth hormone	0	4 (12.9%)	2 (4.7%)	

*Significant difference at* p* < 0.05

Data in table are reported as the median with the range in square brackets, the mean ± standard deviation and a number with the percentage in parenthesis, as appropriate

Univariate analysis using analysis of variance (ANOVA), Kruskal–Wallis test or χ2-test for overall differences at baseline

CKD, Chronic kidney disease; BMI, body mass index; GFR, glomerular filtration rate; ACE, angiotensin converting enzyme; ARB, angiotensin receptor blocker

^a^The study cohort comprised children with CKD, either non-dialysis CKD stage 2–5 patients (CKD group) or renal transplant recipients (CKD-T group), as well as a reference group

^b^Definition urinary albumin ≥20 mg/L

^c^Azathioprine for CKD and tacrolimus and mycophenolate mofetil for CKD-T

^d^Post-hoc testing revealed significant associations between the CKD and CKD-T groups only (*p* < 0.05)

^e^Post-hoc testing revealed significant associations between all three groups (*p* < 0.001)

### Biomarkers of cardiovascular risk at baseline

Markers for mineral metabolism are presented in Table 2. While there was no statistical difference between groups for calcium, phosphate and soluble Klotho, FGF23 and i-PTH levels were higher in both patient groups. The prevalence of hypercalcemia and hyperphosphatemia was only 6.7 and 9.7% in CKD and 2.3 and 4.9% in CKD-T patients, respectively, while elevated i-PTH levels were present in 76% of CKD and 48% of CKD-T patients. Various definitions for high FGF23 levels in pediatric CKD have been published [6, 7], but using the 95th percentile as cutoff [31] in our cohort the prevalence was 60% in CKD and 42% in CKD-T patients, respectively. Low Klotho levels (defined as ≤765 pg/mL) [6] were only present in 6.7% of CKD and 12% of CKD-T patients. Other biomarkers assessed to measure cardiovascular risk were categorized into inflammatory state, lipid profile, anemia and glucose metabolism (see Electronic Supplementary Material).

**Table 2:** Mineral metabolism and cardiac measures at baseline

Baseline mineral metabolism and cardiac measures	Study cohort			*p*-value
	Reference	CKD	CKD-T	
Mineral metabolism (*n* patients)	11	31	43	
Albumin (g/L)	40.5 ± 2.66	38.6 ± 4.09	39.9 ± 2.83	0.29
Calcium^a^ (mmol/L)	2.36 ± 0.10	2.41 ± 0.10	2.41 ± 0.10	0.16
Phosphate (mmol/L)	1.35 ± 0.22	1.47 ± 0.29	1.36 ± 0.23	0.14
i-PTH (ng/L)	41 [28–80]	106 [20–391]	64 [19–195]	<0.001*^b^
FGF23 (RU/ml)	59 [39–82]	175 [68–1225]	114 [45–3809]	<0.001*^b^
FGF23 (*z*-score)	−0.44 [−1.5–0.18]	2.6 [−0.34–12.0]	1.4 [−0.96–43.0]	<0.001*^b^
Klotho (pg/ml)	1746 [671–3438]	1418 [501–3314]	1918 [435–4516]	0.23
Cardiac measures (*n* patients)	9	26	28	
LVMI (g/m^2.7^)	26.7 [18.8–45.7]	34.1 [22.5–53.4]	36.9 [23.9–58.5]	0.03*^c^
cc-TDI e′ (cm/s)	11.5 [9.8–14.1]	11.1 [7.4–13.8]	10.8 [7.8–13.6]	0.26
cc-TDI e′/a′	3.6 [2.4–7.1]	2.9 [1.8–6.3]	2.6 [1.6–4.4]	0.01*^c^
PWD E/TDI e′	7.7 [7.1–9.2]	9.0 [6.7–13.3]	9.1 [6.5–17.5]	0.07

*Significant difference at* p* < 0.05

Data in table are reported as the mean ± SD and the median with the range in square brackets, unless indicated otherwise

Univariate analysis was performed using ANOVA or Kruskal–Wallis test for overall differences at baseline

i-PTH, Intact parathyroid hormone; FGF23 fibroblast growth factor 23; LVMI, left ventricular mass index; PDW, pulse wave Doppler; cc-TDI, color-coded tissue Doppler imaging; e', myocardial relaxation velocity in early diastole﻿, E, passive ventricular filling velocity in early diastole, a′, myocardial velocity due to ﻿﻿﻿﻿atrial contraction in late diastole

^a^Albumin-adjusted calcium

^b^Post-hoc testing revealed significant associations between all three groups (*p* < 0.001)

^c^Post-hoc testing revealed significant associations between the references and CKD-T patients only (*p* < 0.05)

### Longitudinal patterns

In univariate analyses of changes during follow-up, mean GFR was not significantly changed in the CKD group, while mean GFR declined in CKD-T patients, with an annual rate of 2.7 ml/min/1.73 m^2^ (*p* < 0.001). In line with GFR, log FGF23* z*-score did not change significantly in CKD patients (β = 0.12, *p* = 0.08), but increased over time in CKD-T patients (β = 0.21, *p* < 0.001). Also, while the phosphate level decreased by 0.05 (*p* = 0.001) and 0.04 (*p* < 0.001) mmol/L per year in both CKD and CKD-T patients, Klotho and albumin-adjusted calcium levels only decreased in CKD-T patients, by 0.05 log pg/mL (*p* = 0.03) and 0.01 mmol/L (*p* = 0.008) per year, respectively. Further, during the 3-year follow-up period, SBP decreased with 0.15* z*-score per year (*p* = 0.02) in CKD patients, while the diastolic BP* z*-score, BMI* z*-score, inflammatory and lipid profile, hemoglobin level and markers of glucose metabolism remained unchanged in all study participants (data not shown).

### Renal function and the FGF23–Klotho axis

**Fig. 2:**
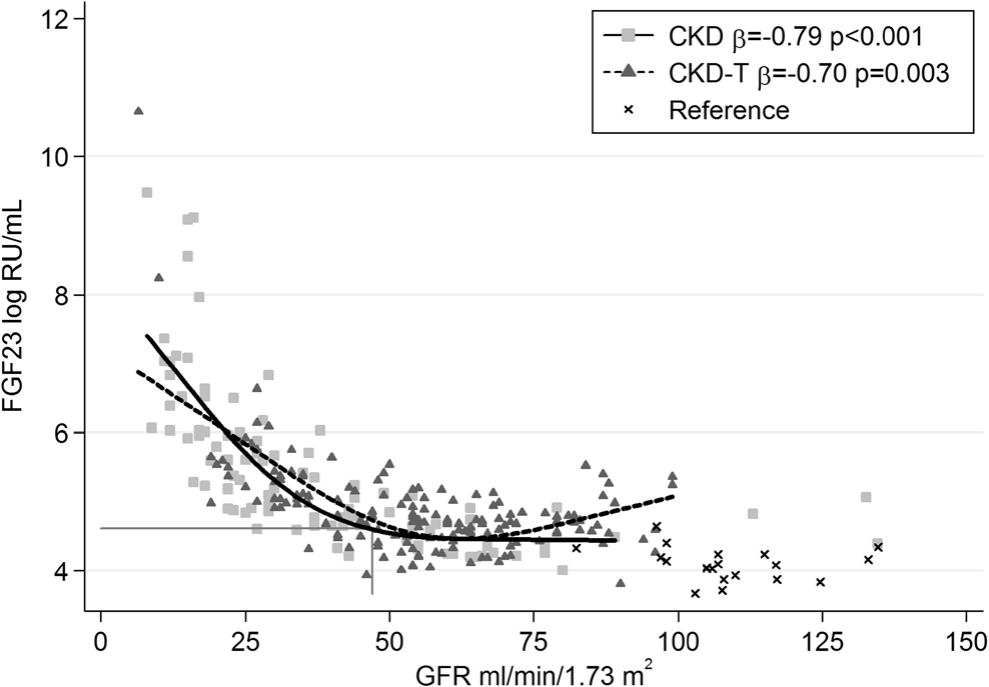
Linear regression analysis for longitudinal correlations between log fibroblast growth factor 23 (*FGF23*) and glomerular filtration rate (*GFR*) using generalized estimating equations (GEE) with an independence covariance matrix. Data are presented graphically using cubic splines with 3 knots due to the non-linear relationship between FGF23 and GFR. *Vertical line* GFR level at which the mean FGF23 is above the upper reference limit (101 RU/mL = 4.62 log RU/mL) for the CKD patients

**Fig. 3:**
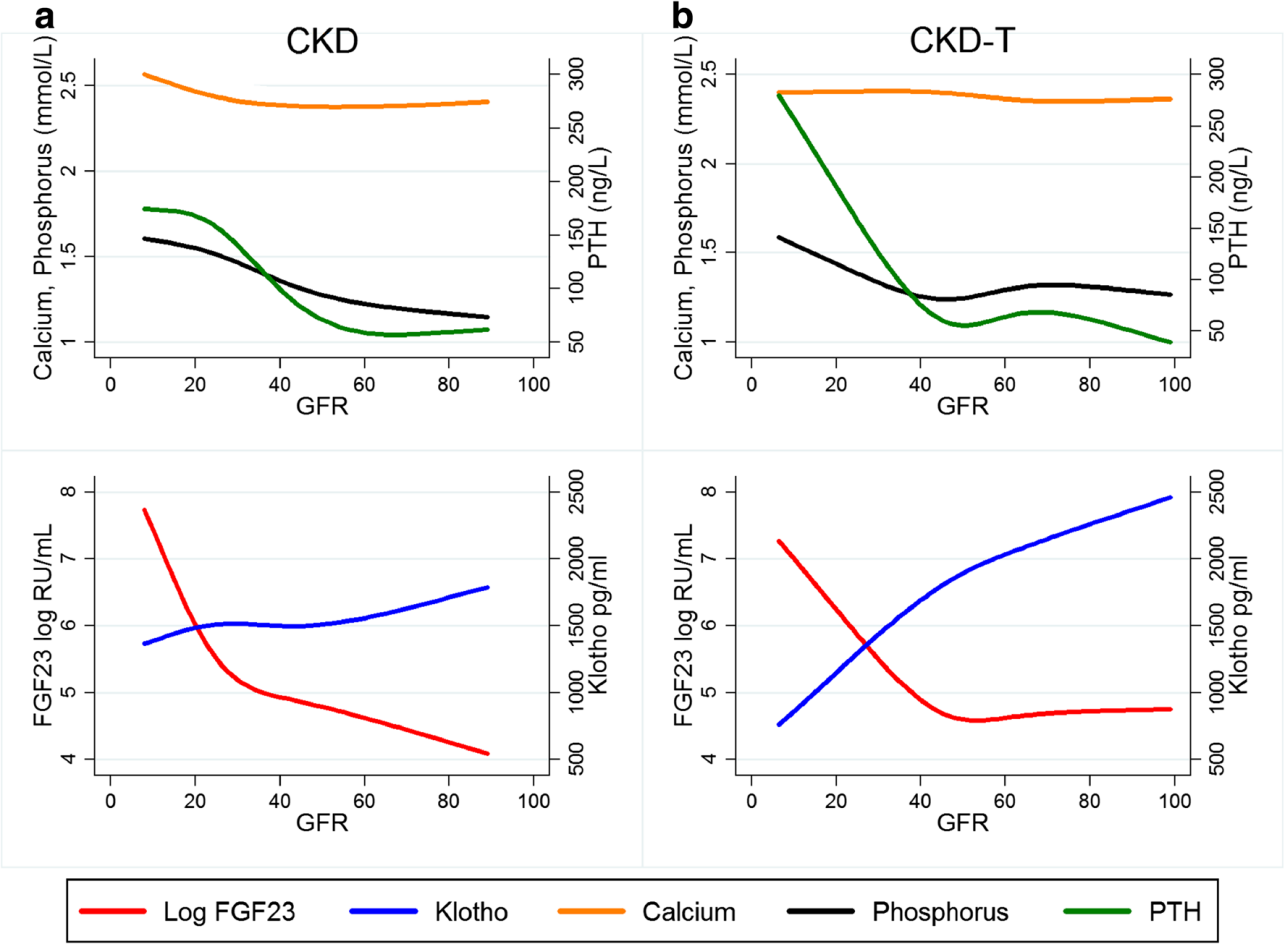
Changes in biomarkers with decreasing GFR values in the CKD and CKD-T patients according to a univariate mixed model with GFR as independent variable and the outcome presented in the* y-axis* as dependent variable. The predictions are made for an individual with a random intercept of zero. GFR is modeled using cubic splines with 4 knots.* PTH* Parathyroid hormone

Log FGF23 was strongly inversely associated with GFR in a non-linear fashion in both CKD (β = −0.79, *p* < 0.001) and CKD-T (β = −0.70, *p* = 0.003) patients, with the threshold for high FGF23 (>101 RU/mL) at a GFR of 47 ml/min/1.73 m^2^ (Fig. 2). To analyze log FGF23, Klotho, albumin-adjusted calcium, phosphate, and i-PTH levels as functions of GFR, we plotted these variables against GFR in both CKD and CKD-T patients (Fig. 3a, b). The GFR at which each substance had changed significantly when compared to the level set at a GFR of 89 ml/min/1.73 m^2^ was determined. In the CKD and CKD-T groups, log FGF23 was noted to have changed significantly at a GFR of 45 and 38 ml/min/1.73 m^2^, i-PTH at a GFR of 33 and 40 ml/min/1.73 m^2^, phosphate at a GFR of 36 and 21 ml/min/1.73 m^2^ and albumin-adjusted calcium at a GFR of 13 and 26 ml/min/1.73m^2^, respectively. Also, Klotho levels decreased significantly relatively early in the CKD progression (GFR 53 ml/min/1.73 m^2^), but only in CKD-T patients. Klotho did not change significantly at any level of GFR in CKD patients.

**Fig. 4:**
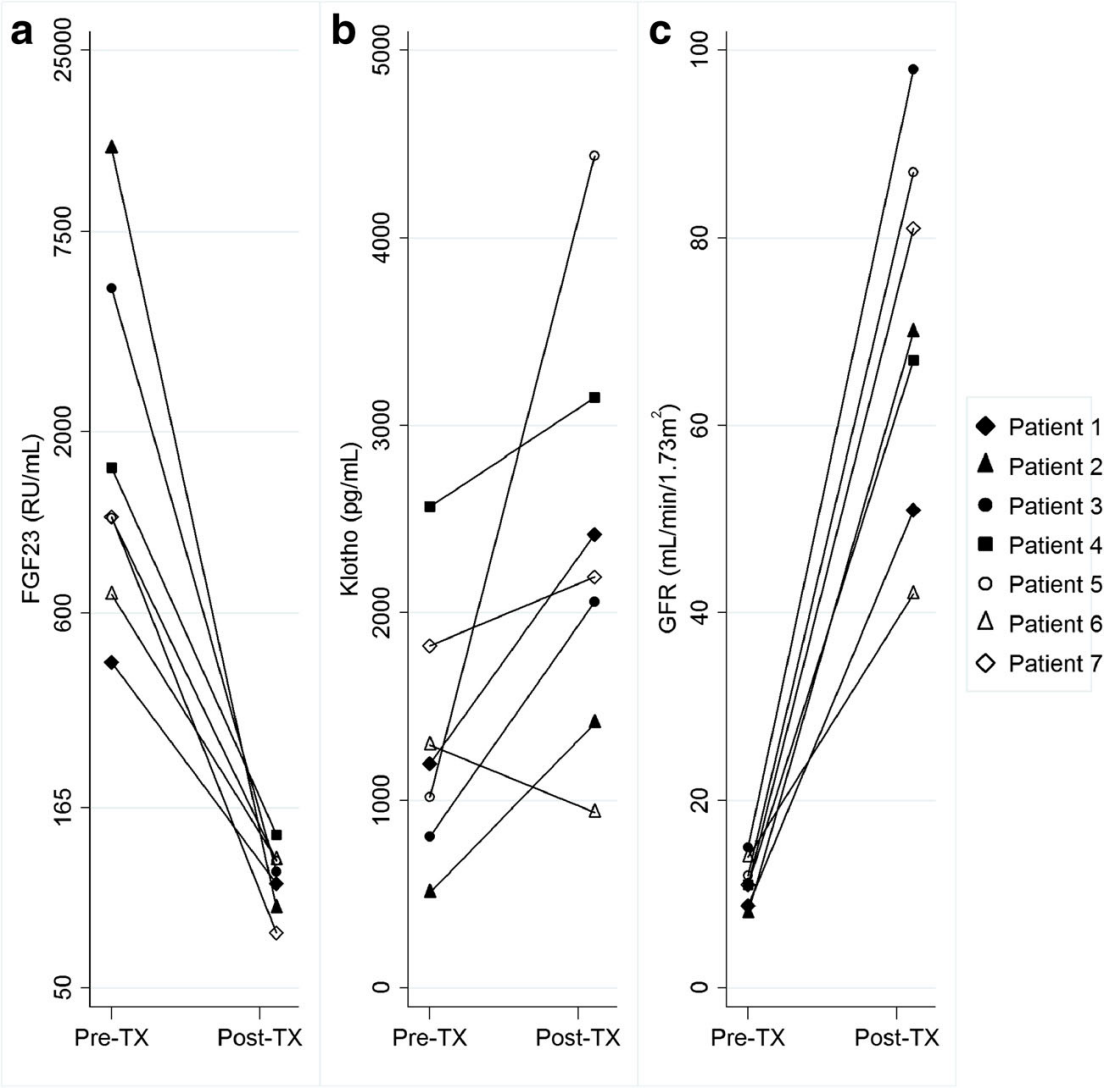
Changes in fibroblast growth factor 23 ( FGF23), Klotho and glomerular filtration rate (GFR) levels in the 7 pediatric patients with chronic kidney disease (CKD) who were transplanted during the follow-up period. Differences were tested before and after renal transplantation (*TX*) using the paired Wilcoxon test: FGF23 *p* = 0.02, Klotho *p* = 0.03, GFR *p* = 0.02. The median post-transplant time was 1.1 (range 0.5–1.1) years

Log FGF23, Klotho and GFR were also assessed in the seven CKD patients who were transplanted during follow-up, comparing levels before and after transplantation (Fig. 4a–c). The post-transplant analyses were made at a median of 1.1 (range 0.5–1.2) years after transplantation. This analysis reveals a marked decline in log FGF23 levels (*p* = 0.02) following renal transplantation, corresponding to the rise in GFR (*p* = 0.02). Similarly, all but one patient showed an increase in log Klotho levels after transplantation (*p* = 0.03). Also, after transplantation, log i-PTH decreased significantly (*p* = 0.03), but albumin-adjusted calcium or phosphate levels did not change significantly (*p* = 0.45 and *p* = 0.13).

### Determinants of FGF23 and Klotho

Table 3 shows data from a multivariable model of potential associations for log FGF23 and log Klotho in all patients (Model 1). Following adjustments, FGF23 exhibited a mean annual increase of 0.11 (*p* < 0.001) log RU/mL. Log FGF23 was positively correlated to phosphate levels in both patient groups (β = 0.97, *p* < 0.001), as were albumin-adjusted calcium levels (β = 1.18, *p* = 0.001). In contrast, GFR was inversely associated with log FGF23 (β = −0.01, *p* < 0.001). Also of interest, the use of vitamin D was associated with increased log FGF23 (β = 0.31, *p* = 0.01). The multivariable model with log Klotho as outcome parameter revealed a significant association to age (β = −0.03, *p* = 0.04) and log FGF23 (β = −0.10, *p* = 0.02).

**Table 3:** Multivariable mixed model of longitudinal associations and predictors of fibroblast growth factor-23 (FGF23) and Klotho

Outcome	Independent variable	MODEL 1^a^ (associations)					MODEL 2^b^ (predictions)				
		β	95% CI	*p* value	Model *p*	*R* ^2^	β	95% CI	*p* value	Model *p*	*R* ^2^
FGF23 (log RU/ml)	Time in study (years)	0.11	(0.06, 0.17)	<0.001	<0.001	0.62	0.09	(0.04, 0.14)	0.001	<0.001	0.54
	GFR (ml/min/1.73 m^2^)	−0.01	(−0.02, −0.006)	<0.001			−0.02	(−0.03, −0.01)	<0.001		
	Phosphate (mmol/L)	0.97	(0.61, 1.33)	<0.001			0.49	(−0.03, 1.00)	0.06		
	Calcium (mmol/L)^c^	1.18	(0.45, 1.91)	0.001			−1.39	(−2.86, 0.08)	0.07		
	Use of vitamin D (yes/no)	0.31	(0.07, 0.54)	0.01			0.52	(0.16, 0.89)	0.005		
Klotho (log pg/ml)	Age at baseline (years)	−0.03	(−0.05, −0.002)	0.04	0.006	0.10	−0.03	(−0.06, −0.003)	0.03	0.002	0.18
	GFR (ml/min/1.73 m^2^)	0.001	(−0.003, 0.004)	0.80			0.01	(0.004, 0.02)	0.002		
	FGF23 (log RU/ml)	−0.10	(−0.18, −0.02)	0.02			0.14	(−0.10, 0.37)	0.25		

All significant variables (*p*-value < 0.05) are listed in the table

CI, Confidence interval; CKD, chronic kidney disease; CKD-T, chronic kidney diseae and transplant; iPTH, intact parathyroid hormone

Linear mixed model showing associations

^a^ (Model 1) and predictors for

^b^ (Model 2) log FGF23 and log Klotho. Model 1 includes: age at baseline (years), time of follow-up from baseline (years), GFR (ml/min/1.7 3 m^2^), phosphate level (mmol/L), albumin-adjusted calcium level (mmol/L), i-PTH level (log ng/L), use of vitamin D (yes/no), patient group (CKD or CKD-T) and FGF23 (log RU/mL) or Klotho (log pg/mL) Model 2 includes all independent variables in Model 1 listed as baseline values to assess true predictors

^c^Albumin-adjusted calcium

### Predictors for FGF23 and Klotho

In a second model (Model 2) possible predictors at baseline were tested against log FGF23 and log Klotho as outcome variables, as shown in Table 3. The only significant predictor for increasing log FGF23 over the follow-up period in both patient groups was low GFR at baseline (β = −0.02, *p* < 0.001). High GFR (β = 0.02, *p* = 0.03) and young age (β = −0.03, 0.03) at baseline predicted higher log Klotho levels longitudinally.

### The FGF23–Klotho axis and cardiovascular findings

The longitudinal importance of high FGF23 and low Klotho on LVMI, cc-TDI e′/a′ and PWD E/TDI e′ was further analyzed. Available echocardiographs for each year are shown in Fig. 1 and baseline data are shown in Table 2. Compared with the reference children, CKD-T patients had the highest baseline LVMI and the lowest cc-TDI e′/a′ (overall *p* = 0.03 and *p* = 0.01 respectively). Table 4 shows results from the multivariable linear mixed model with determinants for LVMI and LV diastolic function. While log FGF23 and FGF23* z*-scores were positively associated with LVMI in univariate analyses in CKD patients (β = 1.7, *p* = 0.01 and β = 1.6, *p* = 0.02, respectively), the results changed following adjustments (β = 1.3, *p* = 0.17 and β = 1.8, *p* = 0.06, respectively). However, both log FGF23 (β = −0.43, *p* = 0.01) and log Klotho (β = 0.44, *p* = 0.006) were significantly associated with worse TDI e′/a′ in CKD-T patients in the multivariate model. Further, there was a positive association between cholesterol and PWD E/TDI e′ (β = 0.44, *p* = 0.03) in CKD patients.

**Table 4:** Multivariable mixed model of longitudinal associations of left ventricular mass index and left ventricular diastolic function in CKD and CKD-T patients

Group	Outcome	Independent variable	Univariate model^a^			Multivariate model^b^				
			Β	95% CI	*p* value	β	95% CI	*p* value	Model p	*R* ^2^
CKD	LVMI (g/m^2.7^)	Age at baseline (years)	−0.56	(−1.1, −0.07)	0.03*	−0.56	(−1.1, −0.01)	0.05*	0.02	0.22
		GFR (ml/min/1.73 m^2^)	−0.08	(−0.15, −0.002)	0.05*	−0.02	(−0.13, 0.08)	0.68		
		FGF23 (log *z*-score)	1.6	(0.22, 3.0)	0.02*^c^	1.8	(−0.07, 3.7)	0.06		
	cc-TDI (e′/a′)	Triglyceride (log mmol/L)	0.41	(−0.06, 0.88)	0.09	0.51	(0.03, 1.00)	0.04*	0.12	
	PWD E/TDI (e′)	Age at baseline (years)	−0.17	(−0.28, −0.07)	0.002*	−0.14	(−0.25, −0.03)	0.01*	<0.001	0.31
		Phosphate (mmol/L)	1.4	(0.24, 1.4)	0.02*	0.75	(−0.59, 2.1)	0.27		
		Cholesterol (mmol/L)	0.60	(0.20, 1.0)	0.004*	0.44	(0.05, 0.83)	0.03*		
CKD-T	LVMI (g/m^2.7^)	BMI (*z-*score)	3.0	(1.3, 4.6)	<0.001*	2.5	(0.75, 4.3)	0.005*	<0.001	0.13
		SBP (*z*-score)	1.6	(0.23, 3.0)	0.02*	1.1	(−0.35, 2.5)	0.14		
	cc-TDI (e′a′)	FGF23, log RU/mL	−0.34	(−0.66, −0.02)	0.04*^d^	−0.43	(−0.77, −0.09)	0.01*	0.007	0.10
		Klotho (log pg/ml)	0.35	(0.06, 0.64)	0.02*	0.44	(0.13, 0.75)	0.006*		
		SBP (*z*-score)	−0.18	(−0.33, −0.03)	0.02*	−0.16	(−0.32, 0.0007)	0.05		
	PWD E/TDI (e′)	Age at baseline (years)	−0.17	(−0.29, −0.04)	0.009*	−0.16	(−0.29, −0.03)	0.02*	0.11	

*CKD* chronic kidney disease, *CKD-T* chronic kidney disease with transplant, *LVMI* left ventricular mas index, *TDI* tissue Doppler imaging, *PWD* pulse wave Doppler, *BMI* body mass index, *SBP* systolic blood pressure, *GFR* glomerular filtration rate, *iPTH* intact parathyroid hormone, *ACE-1* angiotensin converting enzyme, *ARB* angiotensin receptor blocker, *FGF23* fibroblast growth factor 23

*Significant at *p* value < 0.05

^a^In univariate models the following independent variables were tested for an association with the outcome: age at baseline (years), gender, BMI (*z*-score), systolic blood pressure (SBP) (*z*-score), GFR (ml/min/1.73 m^2^), phosphate (mmol/L), albumin-adjusted calcium (mmol/L), use of vitamin D (yes/no), FGF23 (log RU/mL), FGF23 (log* z*-score), Klotho (log pg/ml), i-PTH (log ng/L), hemoglobin (g/L), high-sensitivity C-reactive protein (hsCRP); (﻿log mg/L), cholesterol (mmol/L), triglycerides (log mmol/L), insulin (log μIU/mL), homeostasis model assessment index (HOMA-IR) (log), albuminuria and the use of ACE inhibitors (ACE-I) and/or ARBs. Regarding FGF23 we chose to include log RU/ml or log* z*-score depending on which variable had the best fit in the multivariate model

^b^In the multivariate model, all variables associated with the outcome in univariate analyses (cutoff for *p* value 0.10) were included. Age at baseline, time of follow-up from baseline (years), BMI* z*-score, SBP* z*-score and GFR were forced into the model due to potential confounding

^c^Univariate analyses using log FGF23 RU/ml, β = 1.7 (95% CI 0.35, 3.1,) *p* = 0.01

^d^Univariate analyses using log FGF23* z*-scores: β = −0.16 (95% CI 0.33, 0.008), *p* = 0.06

## Discussion

We have investigated longitudinal changes in FGF23 and Klotho in children with CKD stage 2–5 and in pediatric renal transplant recipients over a time frame of 3 years and examined potential associations of FGF23 and Klotho to measures of cardiac morbidity. The main findings of our study are that FGF23 level was elevated in pediatric CKD and CKD-T patients and that both low baseline GFR and elevated mean levels of phosphate were independent predictors of FGF23. However, our observations that FGF23 increased over time independent of changes in GFR and mineral metabolism indicate that other factors might also be of importance. Interestingly, while elevated FGF23 was associated with increased LVMI in the univariate analyses only, both high FGF23 together with low Klotho level was associated with a worse left ventricular diastolic function (TDI e′/a′) following important adjustments in CKD-T patients.

Recent evidence suggests that FGF23 and Klotho may play important roles in the development of cardiovascular morbidity, both in the general population and in patients with CKD [20, 21, 25, 40]. Both clinical [21] and experimental studies [20, 25, 40] have causally linked FGF23 to cardiac hypertrophy, cardiac dysfunction and congestive heart failure. A direct role of the calcineurin–NFAT (nuclear factor of activated T-cells) signaling pathway, triggered by FGFR-4 activation in cardiac myocytes, has been reported in this context [20]. Our data, which shows associations between increased LVMI, diastolic dysfunction and FGF23, gives clinical support to these data. Still, we and others have not been able to establish a link between FGF23 and cardiac remodeling (LVMI) in pediatric CKD patients receiving conservative treatment [8]. The diagnostic methodology could be one potential factor explaining the discrepancy in our findings. Indeed, Schoenmaker et al. [41] show high variations in LVMI analyses between observers, while TDI has been shown to be a sensitive method to assess cardiac dysfunction [30]. Further, we acknowledge small differences in results where analyses are based on FGF23* z*-score or its raw unadjusted data, which could be another reason for differences between studies. The age effect on FGF23 is still openly debated; consequently, which data are most appropriate to use has not yet been established [31, 42].

Previous reports have shown that the level of FGF23 has increased already at GFR levels ranging from 60 to 69 ml/min/1.73 m^2^ and that they rise exponentially when the GFR falls to <40 ml/min/1.73 m^2^ [7]. We saw a similar trend, with FGF23 starting to increase at a GFR around 60 ml/min/1.73 m^2^, reaching the limit proposed to define high FGF23 levels (101 RU/ml) at GFR 47 ml/min/1.73 m^2^ in CKD patients (Fig. 2). Also in agreement with previous reports [7, 43] the rise in FGF23 observed in our pediatric CKD patients preceded elevations in phosphate. Furthermore, we confirmed results in recent studies [6, 7] in demonstrating a close relationship between phosphate and FGF23 levels in both pediatric CKD and CKD-T patients (Table 3).

Conflicting results have been published on the protective effect of soluble Klotho on cardiovascular events in the general population [44, 45]. Notably, while the prevalence of Klotho deficiency in our CKD-T population was low (12%) in comparison to the prevalence of FGF23 levels of ≥95th percentile (42%), both low Klotho levels and high FGF23 levels were determinants for worse cardiac diastolic function. The synergistic effect of high FGF23 levels and low Klotho levels has been published previously [27]. Similar findings on the association between soluble Klotho and left ventricular diastolic dysfunction in adults has recently been reported [46].

There are few previous reports on soluble Klotho in pediatric CKD [6, 10, 47]. While Klotho levels were reported to be higher in one published study [10], we show levels of soluble Klotho similar to those reported by Wan et al. [6]. Importantly, while there is a close correlation between declining renal function and reduced renal Klotho, there are conflicting results for the correlation between soluble Klotho and GFR [48]. Consistent with these data, even though both our CKD and CKD-T patients had reduced GFR and increased FGF23 levels, soluble Klotho level was within normal range in most patients. Also, while there was a significant increase in Klotho immediately following renal transplantation as renal function was restored, there was no significant association between Klotho and GFR in the adjusted models, suggesting that other factors might influence serum Klotho levels measured with current assays. Previous studies have reported associations between Klotho levels and age, calcium concentration [10], 25-OH vitamin D level [6] and the use of ACE-inhibitors [47]. In our study, Klotho correlated with age, but treatment with ACE-Is and/or ARBs did not have an effect on either FGF23 level or Klotho level, and data on 25-OH vitamin D levels were not available.

There are a number of limitations to this study. First, we do not have information on the levels of 25- or 1.25-(OH) vitamin D. Moreover, we did not assess doses for vitamin D supplementation, but rather we defined “use” or “no use” according to prescribed medications in the patient medical records. Moreover, while data for FGF23 and Klotho over the follow-up period were missing for only 3 and 4% of patients, respectively, for 12% of patients values for the echocardiographic measurements were missing, which might have affected the results on the outcome analysis. Finally, we have an important bias in the CKD group where seven (23%) patients were transplanted during follow-up; thus, those CKD patients with worst renal function dropped out of the follow-up. Nevertheless, to our knowledge our study is the only one to date with a follow-up beyond 1 year in pediatric CKD patients to have examined changes in FGF23 and Klotho levels.

In summary, we report that FGF23 is closely related to phosphate and GFR in pediatric CKD and CKD-T patients and that phosphate and GFR predicts increasing FGF23 levels over time. Additionally, we show that renal transplantation restores FGF23 and Klotho towards normal levels. Finally, while neither FGF23 nor Klotho were significantly associated with LVMI in the adjusted analyses, both high FGF23 and low Klotho were associated with a worse left ventricular diastolic function, as measured by TDI e′/a′ in pediatric CKD-T patients.

## Electronic supplementary material

ESM 1 Supplemental Table (DOCX 15 kb)
