# Supplementary material for: The FGF23–Klotho axis and cardiac tissue Doppler imaging in pediatric chronic kidney disease—a prospective cohort study
Source: Pediatr Nephrol. 2017 Aug 9;33(1):147–57. doi: 10.1007/s00467-017-3766-5 (PMC5700222; doi:10.1007/s00467-017-3766-5)
Supplement: Supplementary file 1 — Supplemental Table (DOCX 15 kb) [file 467_2017_3766_MOESM1_ESM.docx]

**Supplemental Table**

|  | **Reference** | **CKD** | **CKD-T** | **p-value** |
| --- | --- | --- | --- | --- |
| **Inflammatory state** |  |  |  |  |
| hsCRP, mg/L | 0.48 [0.2-1.1] | 0.43 [0.16-8.8] | 0.35 [0.16-19.7] | 0.72 |
|  |  |  |  |  |
| **Lipid profile** |  |  |  |  |
| Triglycerides, mmol/L | 0.61 [0.4-1.0] | 1.0 [0.39-2.7] | 1.2 [0.42-2.8] | **<0.001** |
| Cholesterol, mmol/L | 4.0 ± 0.74 | 4.6 ± 0.99 | 4.0 ± 0.80 | **0.05** |
| LDL, mmol/L | 2.1 ± 0.50 | 2.6 ± 0.85 | 2.1 ± 0.75 | **0.03** |
| HDL, mmol/L | 1.5 ± 0.36 | 1.5 ± 0.39 | 1.4 ± 0.32 | 0.64 |
|  |  |  |  |  |
| **Anemia** |  |  |  |  |
| Haemoglobin, g/L, | 131.9 ± 12.0 | 119.3 ± 13.9 | 122.1 ± 12.0 | **0.02** |
|  |  |  |  |  |
| **Glucose metabolism** |  |  |  |  |
| Glucose, mmol/L | 4.7 [4.1-5.6] | 4.8 [3.6-6.5] | 4.7 [2.7-5.5] | 0.93 |
| Insulin, µIU/mL | 5.3 [2.0-16.9] | 8.3 [2.3-18.6] | 11.1 [2.0-56.6] | 0.11 |
| HOMA-IR | 1.1 [0.37-4.0] | 2.0 [0.37-4.6] | 2.2 [0.24-13.1] | 0.08 |
